# Correction to “Cancer treatment and survivorship statistics, 2025”

**DOI:** 10.3322/caac.70036

**Published:** 2025-09-13

Wagle NS, Nogueira L, Devasia TP, et al. Cancer treatment and survivorship statistics, 2025. *CA Cancer J Clin*. 2025;75(4):308‐340. doi:10.3322/caac.70011

In the “Cancers in children and adolescents” section, the first sentence currently reads: “As of January 1, 2025, it is estimated that 40,260 children (*aged 14 years and older*) and 44,290 adolescents (aged 15–19 years) are living in the United States with a previous cancer diagnosis.” The correct age category for children is “0–14 years” and should read: “As of January 1, 2025, it is estimated that 40,260 children (*aged 0–14 years*) and 44,290 adolescents (aged 15–19 years) are living in the United States with a previous cancer diagnosis.”

The authors apologize for this oversight.

